# Effect of Sodium-Glucose Co-transporter 2 Inhibitors on Bone Metabolism and Fracture Risk

**DOI:** 10.3389/fphar.2018.01517

**Published:** 2019-01-08

**Authors:** Yangli Ye, Chenhe Zhao, Jing Liang, Yinqiu Yang, Mingxiang Yu, Xinhua Qu

**Affiliations:** ^1^Department of Endocrinology, Zhongshan Hospital, Fudan University, Shanghai, China; ^2^Department of Bone and Joint Surgery, Renji Hospital, Shanghai Jiao Tong University School of Medicine, Shanghai, China

**Keywords:** sodium-glucose co-transporter 2, bone turnover, bone microarchitecture, bone strength, bone mineral density, fracture risk, diabetes mellitus, hypoglycemic agents

## Abstract

The effect of anti-diabetic medications on bone metabolism has received increasing attention, considering that type 2 diabetes mellitus is a common metabolic disorder with adverse effects on bone metabolism. Sodium-glucose co-transporter 2 (SGLT2) inhibitors are novel anti-diabetic medications that prevent glucose resorption at the proximal convoluted tubules in the kidney, increasing urinary glucose excretion, and decreasing the blood glucose level. The superiority of SGLT2 inhibitors shows in reducing the glucose level independent of insulin secretion, lowering the risk of hypoglycemia, and improving cardiovascular outcomes. SGLT2 inhibitors have been associated with genital mycotic infections, increased risk of acute kidney injury, dehydration, orthostatic hypotension, and ketoacidosis. Moreover, the effect of SGLT2 inhibitors on bone metabolism and fracture risk has been widely taken into consideration. Our review summarizes the results of current studies investigating the effects of SGLT2 inhibitors on bone metabolism (possibly including increased bone turnover, disrupted bone microarchitecture, and reduced bone mineral density). Several mechanisms are probably involved, such as bone mineral losses due to the disturbed calcium and phosphate homeostasis, as confirmed by an increase in fibroblast growth factor 23 and parathyroid hormone levels and a decrease in 1,25-dihydroxyvitamin D levels. SGLT2 inhibitors might indirectly increase bone turnover by weight loss. Lowering the blood glucose level might ameliorate bone metabolism impairment in diabetes. The effect of SGLT2 inhibitors on bone fractures remains unclear. Evidence indicating the direct effect of SGLT2 inhibitors on fracture risk is lacking and increased falls probably contribute to fractures.

## Introduction

Type 2 diabetes mellitus (T2DM) is a common metabolic disorder that adversely affects bone metabolism and increases the fracture risk (Ahmad et al., 2017; Nilsson et al., 2017). Patients with T2DM are at a higher risk for hip fractures—the most severe among all types of osteoporotic fractures – and limb fractures such as leg or ankle fractures (Forsen et al., 1999; Vestergaard, 2007; Liu et al., 2018). Thiazolidinediones, particularly rosiglitazone but likely also pioglitazone (Schwartz et al., 2015), are associated with increased risk of fractures (Loke et al., 2009; Zhu et al., 2014). In contrast, metformin, sulfonylureas, insulin, dipeptidyl peptidase 4 inhibitors, and glucagon-like peptide 1 agonists appear to not increase the fracture risk (Meier et al., 2016; Wolverton and Blair, 2017). Sodium-glucose co-transporter 2 (SGLT2) inhibitors, a novel class of an-tidiabetic medications, prevent glucose resorption at the proximal convoluted tubules in the kidney, increasing urinary glucose excretion (Chao, 2014). SGLT2 inhibitors exert the same effect in patients with type 1 diabetes mellitus (T1DM), although T1DM is not an indication for SGLT2 inhibitor therapy (Garg et al., 2017). Several SGLT2 inhibitors including canagliflozin, dapagliflozin, and empagliflozin are already available in many countries, whereas others are at various stages of development. Empagliflozin and canagliflozin provide cardiovascular benefits, which may be a class effect (Kosiborod et al., 2017; Neal et al., 2017; Radholm et al., 2018). The superiority of SGLT2 inhibitors in reducing the glucose level independent of insulin secretion, promoting weight loss and lowering blood pressure has been shown (Fadini et al., 2017; Kadowaki et al., 2017; Pfeifer et al., 2017; Softeland et al., 2017; Yale et al., 2017). According to the consensus report by the American Diabetes Association and the European Association for the Study of Diabetes, SGLT2 inhibitors with proven benefits are recommended for diabetic patients with chronic kidney disease or heart failure and atherosclerotic cardiovascular disease (Davies et al., 2018). The reported adverse effects include genitourinary tract infections, dehydration and ketoacidosis (Radholm et al., 2018). Canagliflozin and dapagliflozin are associated with the risk of acute kidney injury in patients with T2DM, which might have a potential effect on bone health. The U.S. Food and Drug Administration has revised the label for canagliflozin to include updates about fracture risk and reduced bone mineral density (BMD) (U.S. Food and Drug Administration, 2016). Taking into account that the issue of bone health and related fracture risk occurrence has led to great economic and social burden, the effect of SGLT2 inhibitors on fractures need to be fully evaluated. We summarize the effects of SGLT2 inhibitors on bone metabolism and their potential mechanisms and discuss their association with fracture risk.

## Effect of T2DM and SGLT2 Inhibitors on Bone Metabolism

### Bone Turnover

Type 2 diabetes mellitus is considered a state of low bone turnover. Bone resorption remained controversial and bone formation appeared to be reduced (Krakauer et al., 1995; Manavalan et al., 2012). But in a meta-analysis, suppressed bone turnover was shown in patients with T2DM (Hygum et al., 2017).

According to current data, the effect of SGLT2 inhibitors on bone turnover varies for different drugs (Table 1). Canagliflozin and ertugliflozin might increase bone resorption, whereas dapagliflozin and empaglflozin might not have an effect on bone turnover. In male diabetic DBA/2J mice, canagliflozin significantly increased the serum concentration of cross-linked C-terminal telopeptides of type I collagen (CTX; RatLaps^TM^), a marker of bone resorption (*p* = 0.0046) (Thrailkill et al., 2016). The significant canagliflozin-induced increase in bone resorption was also observed in another animal experiment (*p* < 0.001) (Thrailkill et al., 2017). However, no statistically significant increase in serum procollagen type 1 N-terminal propeptide (P1NP) was shown (*p* = 0.11).

**Table 1:** Published animal and human studies on the effect of SGLT2 inhibitors on bone metabolism and fractures.

Reference	Subjects and design	Main results (BMD; bone turnover markers; bone microarchitecture; fractures)
**Animals**		
Thrailkill et al., 2016	12-week-old DBA/2J male mice STZ + canagliflozin vs. STZ + vehicle vs. CONT + canagliflozin vs. CONT + vehicle for 10 weeks	**Bone microarchitecture:** Bone strength in femur and the sixth lumbar vertebra were impaired. Detrimental drug-induced effect on trabecular bone was seen. **Bone turnover markers:** Serum levels of CTX increased.
Thrailkill et al., 2017	12-week-old DBA/2J male mice STZ + canagliflozin vs. STZ + INS vs. STZ + canagliflozin + INS vs. STZ + no treatment vs. non-diabetic control mice for 9 weeks	**Bone microarchitecture:** Bone microarchitecture and bone strength in femur and vertebra were significantly impaired. **Bone turnover markers:** Serum levels of CTX increased but serum levels of P1NP did not change.
Suzuki et al., 2014	Male Wistar rats and KKAy mice 9 weeks diets containing tofogliflozin	**Bone microarchitecture:** No changes of bone mass by microcomputed tomography at week 8 were shown.
**Human**		
Ljunggren et al., 2012	182 metformin-treated T2DM patients Dapagliflozin 10 mg vs. placebo added to metformin for 50 weeks	**Bone turnover markers:** No significant changes of serum levels of P1NP and CTX were shown over 50 weeks. **BMD:** BMD levels were not change significantly. **Fractures:** No incidence of fractures was reported.
Bilezikian et al., 2016	716 patients with T2DM aged 55–80 years Canagliflozin 100 mg vs. 300 mg vs. placebo for 104 weeks	**Bone turnover markers:** Serum levels of CTX and OC increased at week 26 and week 52. **Bone microarchitecture:** Bone strength at the spine and femoral neck did not change at week 52. **BMD:** No changes in volumetric BMD at the spine and femoral neck at week 52. BMD levels decreased in lumbar spine and total hip. No significant change at distal forearm and femoral neck at week 104.
Rosenstock et al., 2018	621 patients with T2DM treated with metformin Ertugliflozin 5 mg vs. 15 mg vs. placebo with 26 weeks treatment	**BMD:** Ertugliflozin had no adverse impact on BMD at week 26. **Bone turnover markers:** Mean serum levels of CTX increased in ertugliflozin groups in a dose-related manner, but mean serum levels of P1NP were similar to baseline at week 26.
Ruanpeng et al., 2017	A meta-analysis of 20 studies including 8,286 patients treated with SGLT2 inhibitors	**Fractures:** Increased risk of bone fracture with SGLT2 inhibitors was not observed.
Tang et al., 2016	A meta-analysis of 38 randomized controlled trials (canagliflozin, dapagliflozin and empagliflozin) involving 30384 patients in 24–160 weeks follow-up	**Fractures:** No harmful effect of SGLT2 inhibitors on fractures.


*BMD, bone mineral density; STZ, streptozotocin; CONT, control; INS, insulin; P1NP, procollagen type 1 N-terminal propeptide; T2DM, type 2 diabetes mellitus; CTX, C-terminal cross-linking telopeptides of type I collagen; SGLT2, sodium-glucose co-transporter 2.*

With respect to human studies, CTX was shown to modestly increase in a phase II clinical studies on canagliflozin (*n* = 451); nonetheless, no significant changes in P1NP and osteocalcin were observed after 12-week canagliflozin treatment (Rosenstock et al., 2012). In their double-blind, placebo-controlled phase III study (*n* = 621), Bilezikian et al. showed that CTX significantly increased with canagliflozin treatment. Furthermore, a statistically significant relationship was found between increases in CTX and weight loss (*P* < 0.001 at week 26) (Bilezikian et al., 2016). No effects on bone resorption or formation were noted after 50 and 102 weeks of treatment with dapagliflozin (Ljunggren et al., 2012; Bolinder et al., 2014). Similar results were reported with empagliflozin (Kohler et al., 2017). As diabetes may be associated with a reduction in enzymatic cross-links, CTX may underestimate bone resorption in diabetic patients (Saito et al., 2006; Saito and Marumo, 2010). Thus, it remains unclear whether increased bone resorption clinically occurs following treatment with different SGLT2 inhibitors.

### Bone Microarchitecture and Bone Strength

T2DM is associated with deficits in the trabecular and cortical bone microarchitecture in the femur and axial skeleton in animal studies (Thrailkill et al., 2016). Unfavorable cortical bone microarchitecture (increased cortical porosity) at the distal radius (Burghardt et al., 2010; Yu et al., 2015) and its potential detrimental effects on bone strength (Farr et al., 2014) were observed in postmenopausal women with T2DM. Bone strength at the cortical-rich midshaft of the radius was reduced in oldegr men with T2DM despite no difference in cortical volumetric BMD (Petit et al., 2010).

Canagliflozin might have detrimental effects on the bone microarchitecture, which could be explained by the diabetes-related reduction in bone structural strength and bone toughness (Table 1). In male diabetic DBA/2J mice, treatment with canagliflozin for 10 weeks adversely affected the cortical and trabecular bone microarchitecture, diminishing bone strength in the femur, and vertebrae. In non-diabetic mice, canagliflozin decreased the trabecular bone volume fraction, trabecular number, and trabecular tissue mineral density in the femur and increased trabecular spacing (*p* < 0.0001) (Thrailkill et al., 2016). Another animal study noted that the reduction in bone structural strength and bone toughness in the femur and the vertebral body was significantly explained by glycemic control. Moreover, SGLT2 was not detected in any of the osteoblast or osteoclast cell lines (Thrailkill et al., 2017). We speculate that canagliflozin has detrimental effects on the bone microarchitecture. Nevertheless, there is a lack of human studies on changes in the bone microarchitecture. Relevant preclinical or clinical data clarifying how SGLT2 inhibitors affect bone matrix mineralization and collagen fiber distribution are also required.

### Bone Mineral Density

Bone mineral density may remain unchanged or may either decrease or increase in patients with T2DM (Schwartz et al., 2005; Petit et al., 2010; Zhou et al., 2010). Some studies show individuals with T2DM tend to have a higher BMD (Vestergaard, 2007). Increased bone loss at the femoral neck has been observed in diabetic white women although they have the higher baseline BMD (Schwartz et al., 2005). Increased BMD has been associated with body mass index, whereas insulin resistance has been associated with low bone turnover (Laurent et al., 2016).

Canagliflozin results in a reduction in total hip BMD (Table 1), and this might be partly explained by weight loss (Bilezikian et al., 2016). Based on data from a placebo-controlled, phase III clinical trial that included patients with T2DM aged 55–80 years (*n* = 716), treatment with canagliflozin for 104 weeks was associated with a reduction in BMD in the total hip, but not at the femoral neck, lumbar spine, or distal forearm. Change in body weight was a statistically significant covariate (*P* < 0.05) and appeared to explain approximately 40% of the observed difference in total hip BMD (Bilezikian et al., 2016). In addition, reduced estradiol levels might partly contribute to BMD loss, as canagliflozin treatment has been shown to reduce estradiol levels in female patients, which has not been fully elucidated. Rosenstock et al. reported that ertugliflozin had no adverse effect on BMD at the lumbar spine, femoral neck, total hip, and distal forearm regions at week 26 in either the overall population or the cohort of postmenopausal women for ≥3 years of follow-up (Rosenstock et al., 2018). Similarly, dapagliflozin had no significant differences in BMD in the lumbar spine, femoral neck, and total hip after 50 and 102 weeks (Ljunggren et al., 2012; Bolinder et al., 2014).

In an animal model, change in bone mass was not observed after administration of tofogliflozin for 9 weeks (Suzuki et al., 2014). Weight loss could only partly explain the decrease in BMD; hence, further studies are required to elucidate whether SGLT2 inhibitors have an effect on changes in BMD after treatment with different drugs.

### Potential Mechanism of SGLT2 Inhibitors on Bone Metabolism

**FIGURE 1:**
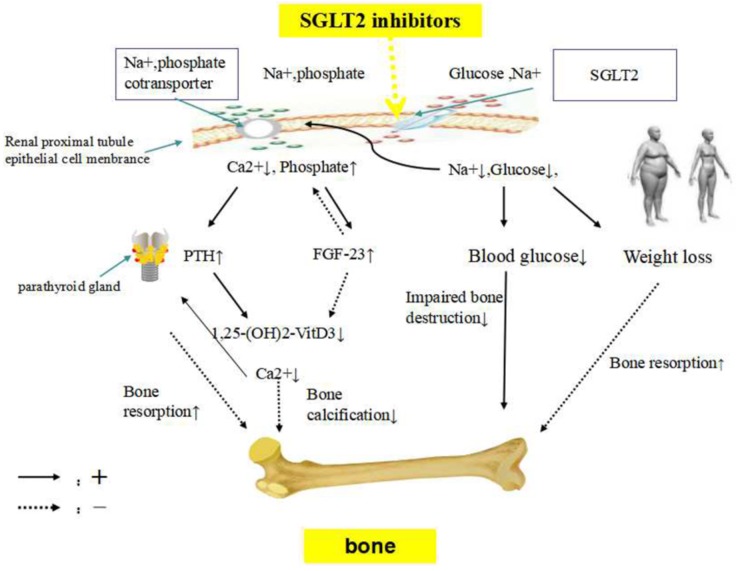
The potential mechanism of SGLT2 inhibitors on bone metabolism. SGLT2 inhibitors, sodium-glucose co-transporter 2 inhibitors; FGF23, fibroblast growth factor 23; PTH, parathyroid hormone. SGLT2 inhibitors inhibit sodium and glucose co-transporter, enhancing phosphate transportation via sodium-phosphate co-transporter in the renal proximal tubules. Sodium loss would lead to increased phosphate resorption and urinary calcium excretion. FGF23, which regulates systemic phosphate homeostasis and vitamin D metabolism, might be stimulated by higher serum phosphate concentration. Consequently, elevations in serum FGF23 results in phosphaturia and inhibits the production of 1,25-dihydroxyvitamin D production, to maintain phosphate balance. A decrease in 1,25-dihydroxyvitamin D concentrations reduces calcium absorption from the gastrointestinal tract, probably impairing skeletal mineralization. The reduction in calcium concentration due to urinary calcium excretion leads to the development of secondary hyperparathyroidism, PTH secretion is stimulated. SGLT2 inhibitors might indirectly increase bone turnover by weight loss. Lowing the blood glucose level might ameliorate bone metabolism impairment in diabetes.

There are probably several mechanisms for the effect of SGLT2 inhibitors on bone metabolism, including the disordered calcium and phosphate homeostasis due to the inhibition of sodium–glucose co-transporter. This contributes to increased parathyroid hormone (PTH) levels and reduced 1,25-dihydroxy vitamin D levels, affecting bone metabolism. SGLT2 inhibitors might indirectly increase bone turnover by weight loss and ameliorate bone metabolism impairment in diabetes by reducing the blood glucose level (Figure 1).

### Calcium and Phosphate Homeostasis

Urinary calcium loss (*p* = 0.003) and fibroblast growth factor 23 (FGF23) levels (*p* = 0.046) were observed to have increased in canagliflozin-treated diabetic mice (Thrailkill et al., 2016). Small increases in phosphate (0.06 mmol/L) from baseline were detected with dapagliflozin at week 50 (Kohler et al., 2017). Both glucose and phosphate are reabsorbed by sodium-dependent cotransporters that compete for the sodium electrochemical gradient (Curthoys and Moe, 2014). SGLT2 inhibitors might inhibit sodium-glucose co-transporter, enhancing phosphate transportation via sodium-phosphate co-transporter in the renal proximal tubules (Taylor et al., 2015). Sodium loss would lead to increased phosphate re-absorption and urinary calcium excretion. Additionally, FGF23, which regulates phosphate homeostasis and vitamin D metabolism (Quarles, 2012), might be stimulated by higher serum phosphate concentration. Consequently, elevation in serum FGF23 concentration results in phosphaturia and inhibits the production of 1,25-dihydroxyvitamin D, to maintain the phosphate balance (Burnett et al., 2006). A decrease in 1,25-dihydroxyvitamin D concentrations reduced calcium absorption from the gastrointestinal tract (Masuyama, 2014), probably impairing skeletal mineralization. The reduction in calcium concentration due to urinary calcium excretion leads to the development of secondary hyperparathyroidism. PTH secretion is stimulated. Recently, Blau et al. have confirmed that after canagliflozin administration, phosphate, FGF23, and PTH levels increase, whereas 1,25-dihydroxyvitamin D level decreases, which might have detrimental effects on bone health (Blau et al., 2018). Canagliflozin treatment had been shown to reduce the expression of 25-hydroxyvitamin D-1 alpha hydroxylase (CYP27B1) mRNA by 50% in diabetic mice (*p* = 0.012) (Thrailkill et al., 2016).

The observed increase in phosphate, FGF23, and PTH levels and decrease in 1,25-dihydroxyvitamin D levels were small (Kohler et al., 2017) and were noted within the first few hours/day after administration (Blau et al., 2018). Whether these changes are sustained after long-term SGLT2 inhibitor treatment remains unknown.

### Weight Loss

Sodium-glucose co-transporter 2 inhibitors might indirectly increase bone turnover by weight loss. SGLT2 inhibitors have been reported to induce modest weight loss. Weight loss leads to a statistically significant increase in osteocalcin and CTX concentrations, suggesting that weight loss increases bone turnover. Weight loss also decreases total hip BMD but not spinal BMD (Zibellini et al., 2015). According to a published study, approximately 40% of the reduction in total hip BMD with canagliflozin treatment was associated with weight loss. Moreover, an increase in bone resorption was determined to be correlated with the degree of weight loss (CANA 100 mg *p* = 0.007; CANA 300 mg *p* = 0.016) (Bilezikian et al., 2016).

Adiponectin and leptin, which are produced by adipose tissues, exert some effects on osteoblasts and osteoclasts (Tamura et al., 2007; Williams et al., 2009). A positive correlation was shown between the adiponectin/leptin ratio and osteocalcin in obese adolescents, indicating to ameliorate negative influence on bone turnover (Campos et al., 2018). Clinically, a study on SGLT2 inhibitors in obese non-diabetic individuals showed that remogliflozin etabonate significantly reduced the leptin/adiponectin ratio after 8 weeks (Napolitano et al., 2014). Comprehensive data on changes in energy metabolism after fat loss or on the separate effects of SGLT2 inhibitors on leptin and adiponectin are not available. Furthermore, some findings suggest that decreased body weight regulates bone mass independent of leptin (Jansson et al., 2018). It remains unknown how SGLT2 inhibitors affect the function of osteoblasts and osteoclasts. The relationship between adiponectin/leptin ratio and osteocalcin might be helpful to clinically represent alteration in bone turnover.

### Reducing Blood Glucose Level

Sodium-glucose co-transporter 2 inhibitors might ameliorate bone metabolism impairment related to advanced glycation end products (AGEs) by reducing the blood glucose level in diabetic patients. High level of AGEs, which stiffen bone collagen, is adversely related to bone metabolism in the cortical and cancellous bone and is directly associated with reduced osteoblast number and function (Rico et al., 1989; Leslie et al., 2012). Activation of the receptor for AGEs in bone-derived cells can enhance the production of inflammatory cytokine and reactive oxygen species, resulting in a vicious cycle of chronic inflammation and increased bone resorption (Hein, 2006). Reactive oxygen species directly affects differentiation and survival of osteoclasts, osteoblasts, and osteocytes (Manolagas, 2010). A Japanese study showed that SGLT2 inhibitors attenuated the effect of AGEs and suppressed oxidative stress and inflammatory reactions in the kidneys of type 1 diabetic rats (Ishibashi et al., 2016). A high glucose level impairs the function of osteoblastic cells, decreasing bone formation (Inaba et al., 1995). AGE levels in diabetic patients increase as a result of hyperglycemia and oxidative stress. Improvement in glucose control decreases bone turnover and provides protection against bone loss (Okazaki et al., 1997). Decreases in serum hemoglobin A1c and fasting plasma glucose levels have been clinically confirmed, which are consistent with reports for SGLT2 inhibitors (Scheerer et al., 2016; Shyangdan et al., 2016; Zaccardi et al., 2016; Rosenstock et al., 2018). Detrimental changes in bone microarchitecture and bone strength related to diabetes were improved after canagliflozin and insulin combination therapy (Thrailkill et al., 2017). Thus, it has been proposed that SGLT2 inhibitors might decrease the blood glucose level, ameliorating the condition related to AGEs in diabetic patients, and might play an important role in bone metabolism.

## Effect of SGLT2 Inhibitors on Fracture Risk

The effect of SGLT2 inhibitors on fracture risk remains controversial, and evidence indicating the direct effect of SGLT2 inhibitors on fracture risk is lacking (Table 1). A meta-analysis of 38 randomized controlled trials (*n* = 30384) showed that SGLT2 inhibitors were not significantly associated with an increased fracture risk. Compared with placebo, canagliflozin [odds ratio (OR), 1.15; 95% confidence interval (CI), 0.71 to 1.88], dapagliflozin (OR, 0.68; 95% CI, 0.37 to 1.25), and empagliflozin (OR, 0.93; 95% CI, 0.74 to 1.18) were not significantly associated with an increased fracture risk. No differences in the incidence of fractures were observed among these three SGLT2 inhibitors (Tang et al., 2016). In a meta-analysis including 20 studies with 8,286 patients, SGLT2 inhibitors were also not associated with an increased fracture risk (Ruanpeng et al., 2017). In addition, fracture was only mentioned as an event, and it has not been reported that the fracture risk increased with empagliflozin treatment at doses of 10 or 25 mg/day (Haring et al., 2013; Roden et al., 2013; Barnett et al., 2014; Rosenstock et al., 2014; Suzuki et al., 2014; Kovacs et al., 2015). According to a pooled analysis of phase I-III clinical trials on empagliflozin, fractures were similar among treatment groups (1.7, 1.6, and 1.4 per 100 patient-years in placebo, 10 mg/day empagliflozin, and 25 mg/day empagliflozin, respectively) (Kohler et al., 2017). In participants with chronic kidney disease, fractures were not increased with empagliflozin treatment (Barnett et al., 2014).

In contrast, Kohan et al. reported fractures at sites such as the toes and patellae, which might be more likely related to falls, with dapagliflozin treatment in patients with moderately impaired renal function. The review noted in one trial that 10 mg dapagliflozin experience more fractures compared with lower doses (2.5 and 5 mg/day) [9.4% (8/85) of patients receiving 10 mg/day dapagliflozin and 6.0% (5/83) of patients receiving 5 mg/day dapagliflozin] over 104 weeks of follow-up, whereas no fractures were reported in patients receiving placebo (Kohan et al., 2014). In addition, in patients with normal to mildly impaired renal function, dapagliflozin has no effect on markers of bone formation and resorption or on BMD (Ljunggren et al., 2012).

Groups treated with dapagliflozin had higher rates of neuropathy and orthostatic hypotension, more likely resulting in an accidental fall (Kohan et al., 2014). Owing to the mechanism of SGLT2 inhibitors at the proximal convoluted tubules in the kidney, sodium concentration declines with increasing excretion. Postural hypotension due to volume depletion might lead to increased risk of falls, resulting in fractures. Moreover, postural dizziness, orthostatic hypotension, and syncope were also reported with canagliflozin treatment (Weir et al., 2014). The incidence of hypoglycemia was higher with canagliflozin treatment in conjunction with insulin therapy (Neal et al., 2015), which is also associated with a higher prevalence of falls in patients with T2DM. In addition, patients with diabetic complications are at increased risk for falls. Increased falls contribute to a higher fracture risk. Of note, blood glucose control for the prevention of diabetic complications must be balanced against the negative effects of hypoglycemia. Assessment of fall risk and appropriate measures to prevent falls should be performed during canagliflozin treatment.

A pooled analysis of nine studies including the Canagliflozin Cardiovascular Assessment Study (CANVAS) (*n* = 4327) and eight non-CANVAS studies (*n* = 5867) assessed the incidence of fractures after canagliflozin treatment. In the CANVAS, patients had an elevated cardiovascular disease risk, lower estimated glomerular filtration rate (eGFR), longer mean exposure, older age, and greater use of diuretics. The incidence of fractures increased with canagliflozin versus placebo across subgroups in the overall population. In the CANVAS, the incidence of fractures was statistically significantly higher in the pooled canagliflozin group than in the placebo group (4.0% versus 2.6%), independent of dosage. The increased fractures occurred in the lower and upper limbs. However, in the pooled analysis of eight non-CANVAS studies, the incidence of fractures was similar for canagliflozin and non-canagliflozin (Watts et al., 2016). Firstly, the increased fractures occurred in the distal parts of the upper and lower extremities, which might be related to increased fall. In the CANVAS, a sensitivity analysis excluding fractures not associated with osteoporosis or skeletal fragility showed that the incidence of fractures was no longer significantly higher with canagliflozin treatment. As previously mentioned, both T2DM and canagliflozin treatment have detrimental effects on the bone microarchitecture. There is a lack of evidence indicating the direct effect of canagliflozin on fractures. Secondly, increased fractures occurred within the first few weeks after canagliflozin treatment, independent of dosage. Moreover, adverse effects related to volume depletion occurred earlier with canagliflozin treatment at a dose of 300 mg in the CANVAS. It seems that fractures might be more likely related to volume depletion. However, syncope or presyncope was not reported among patients just prior to or within 30 days of experiencing fractures. In addition, the bone fracture rate was similar across treatment groups, although the incidence of adverse effects related to volume depletion was higher with 10 and 25 mg empagliflozin than with placebo in older patients (3.2 and 3.0 versus 2.3/100 patient-years) (Kohler et al., 2017). It remains unclear whether the increased fractures are specific to canagliflozin treatment.

## Conclusion

The results of current studies focusing on the effect of SGLT2 inhibitors on bone metabolism and fractures are controversial. These studies have reported increased bone resorption, disrupted bone microarchitecture, and reduced total hip BMD after canagliflozin treatment in patients with T2DM. Whether these are consistent across individual drugs in the class and are indirect actions of SGLT2 inhibitors remains unclear. Evidence confirming the effect of long-term treatment with SGLT2 inhibitors on fractures is lacking; increased falls probably contribute to fracture risk. SGLT2 inhibitors are novel oral anti-diabetic medications in China, and clinical data are still insufficient to evaluate the effect on fractures. Taking into account that the issue of bone health and related fracture risk occurrence resulted in great economic and social burden, assessment of fall risk and appropriate measures to prevent falls should be employed during canagliflozin treatment while waiting for more clinical evidence. As fractures do not occur in the majority of patients receiving SGLT2 inhibitor therapy, the clinical importance of 1,25-dihydroxyvitamin D and PTH levels, and bone turnover markers, requires further investigation. BMD measurement, assessment of vertebral fractures or osteoporosis treatment should be considered in patients with a history of previous fractures or in elderly patients. As current clinical trials were mostly not designed to examine the effects of these drugs on bone metabolism or fracture risks and the population was not specifically selected to include patients with high fracture risk, more studies are needed.

## Author Contributions

All authors contributed equally to the writing, revision, and editing of this manuscript.

## Conflict of Interest Statement

The authors declare that the research was conducted in the absence of any commercial or financial relationships that could be construed as a potential conflict of interest.
